# Evaluating The Predictive Reliability of Neural Networks in Psychological Research With Random Datasets

**DOI:** 10.1177/00131644241262964

**Published:** 2024-07-25

**Authors:** Yongtian Cheng, K. V. Petrides

**Affiliations:** 1 University College London (UCL), London, UK

**Keywords:** predictive result, supervised neural network, decision error, Balanced accuracy, Monte Carlo simulation

## Abstract

Psychologists are emphasizing the importance of predictive conclusions. Machine learning methods, such as supervised neural networks, have been used in psychological studies as they naturally fit prediction tasks. However, we are concerned about whether neural networks fitted with random datasets (i.e., datasets where there is no relationship between ordinal independent variables and continuous or binary-dependent variables) can provide an acceptable level of predictive performance from a psychologist’s perspective. Through a Monte Carlo simulation study, we found that this kind of erroneous conclusion is not likely to be drawn as long as the sample size is larger than 50 with continuous-dependent variables. However, when the dependent variable is binary, the minimum sample size is 500 when the criteria are balanced accuracy ≥ .6 or balanced accuracy ≥ .65, and the minimum sample size is 200 when the criterion is balanced accuracy ≥ .7 for a decision error less than .05. In the case where area under the curve (AUC) is used as a metric, a sample size of 100, 200, and 500 is necessary when the minimum acceptable performance level is set at AUC ≥ .7, AUC ≥ .65, and AUC ≥ .6, respectively. The results found by this study can be used for sample size planning for psychologists who wish to apply neural networks for a qualitatively reliable conclusion. Further directions and limitations of the study are also discussed.

Neural networks (NNs) are a set of machine learning algorithms inspired by the structure and function of the human brain (Lawrence, 1993). In NNs, interconnected neurons process data by applying adjustable weights and activation functions, enabling the NN to learn and make predictions. NNs with sufficient neurons can fit any complex model with enough iterations (Cybenko, 1989). This ability allows NNs to exhibit exceptional performance in diverse tasks, such as natural language processing (NLP; Zalake & Naik, 2019), image classification (Rawat & Wang, 2017), and psychological research (e.g., Mariani et al., 2022; Martinez-Ramon et al., 2022; Ritter et al., 2017).

In psychology, supervised NNs are often employed to explore patterns between independent variables (IVs) and dependent variables (DVs) (Allahyari & Roustaei, 2022; Cui et al., 2024; Darvishi et al., 2017; Koorathota et al., 2021; Witten et al., 2005). When a fitted supervised NN model identifies IVs that can partially predict a DV, it is generally concluded that they have predictive power on the DV using supervised NN. For example, Marshall and English (2000) applied a supervised NN using various ordinal variables as IVs to assess risk in child-protective services, finding that the Washington Risk Assessment Matrix (Caldwell et al., 1993) could partially predict caregiver risk behaviors, such as dangerous acts and substance abuse.

Similarly, Khan et al. (2019) demonstrated that mobile payment habits could be predicted by Big Five personality traits. Zeinalizadeh et al. (2015) found bank customer satisfaction can be predicted by a psychometric scale proposed in their study by supervised NN. A trending application of neural network models is in educational psychology (Chavez et al., 2023; Noetel et al., 2023; Sandoval-Palis et al., 2020). For example, Psyridou et al. (2024) found various features such as the capability of students on different tasks and psychometric measurement results that can predict math learning difficulty by supervised NN. Pavlekovic et al. (2010) extracted the most vital IVs to predict the gift of mathematical gift of students from various mathematical tasks by supervised NN. Pei (2022) evaluated, predicted, and analyzed the mental health status of contemporary college students based on an NN model.

For ease of discussion, NN will refer specifically to supervised NNs in the following paragraphs. The conclusion provided by supervised NNs is commonly a statement that DV(s) can be partially predicted by IVs in a general population. Meanwhile, the conclusion usually focuses on the accuracy of the prediction, and this performance of the model is viewed as the estimation of the performance of the model on a general population (Dwyer et al., 2018).

Before the integration of NNs into psychology, and even today, psychologists have predominantly used null hypothesis significance testing (NHST) to determine the predictive relationship between IVs and DVs (Cumming, 2014). In NHST, the null hypothesis asserts that there is no relationship between IVs and DVs in regression—a method frequently used in psychological prediction (Frost, 2017). NHST carries the risk of committing a Type-I error, where a true null hypothesis is incorrectly rejected (Adusah & Brooks, 2011; Austin & Brunner, 2004). Unlike NHST, NNs focus solely on the model’s prediction performance, yet they can still present inflated results (Gavrilov et al., 2018; Ying, 2019).

This case raises concerns about the potential for researchers to erroneously infer a relationship between IVs and DVs using NNs, even when no such relationship exists. When traditional regression models fail to predict a DV due to the absence of a linear relationship with IVs, researchers often turn to NNs. Although NNs have demonstrated superior predictive power in certain contexts (Darvishi et al., 2017; Koorathota et al., 2021; Lin et al., 2022), this success might be misleading. Researchers may erroneously conclude the existence of complex nonlinear relationships between the IVs and DV, even when such relationships are absent in the broader population. This misinterpretation, termed “decision error” (DE), highlights a critical concern with NNs. A DE arises when the predictive performance of an NN falsely suggests the IVs’ ability to predict the DV within the population. The criteria for identifying a DE, discussed further below, involve carefully evaluating the NN’s predictive performance and its generalizability.

This study investigates the risk of DE in psychological research using NN models. We aim to assess how often NNs can falsely indicate acceptable prediction accuracy in scenarios where no true relationship exists between IVs and DVs. To do this, we will conduct a Monte Carlo simulation, exploring various conditions to estimate the likelihood of such misleading outcomes. The choice of a Monte Carlo simulation allows for a comprehensive analysis across a wide range of hypothetical scenarios, thereby providing a robust estimation of NN performance in the absence of real IV–DV relationships.

The rest of the case will be organized as follows. First, we will provide an introduction to a typical design of the NN model fitting with an explanation of why the model performance of NN is at risk of DE. Then, we will provide the design of this simulation study to estimate the probability of this risk. After that, we will report the simulation result in a Results section. Finally, a discussion section will be provided with suggestions for psychologists using NNs.

## Reason for NN to Commit DE

To evaluate the risk of NNs leading to DE, it is crucial to understand the typical processes involved in NN model fitting and performance assessment. Overfitting is a common challenge in various supervised machine learning methods, including NNs, characterized by models performing well on training data but poorly on unseen data from the broader population (Ying, 2019). This section introduces the causes of overfitting in NN model fitting, discusses dataset division as a strategy employed by computer scientists to mitigate this issue, and explains how researchers estimate NN model performance in their studies. Despite these efforts, the possibility of committing a DE remains.

NNs, especially those of sufficient complexity, can model any relationship between IVs and DVs, be it linear or nonlinear (Cybenko, 1989). A common issue is over-parameterization, where NNs have more neurons than necessary (Allen-Zhu et al., 2019), enabling them to memorize specific IV–DV combinations in the training dataset. This capability, however, often results in subpar performance when applied to data representing the broader population. In the next paragraph, we will discuss why this phenomenon occurs and highlight the persistent risk of DE in psychological research using NNs.

In datasets containing pairs of IVs and DVs from a population, there are two distinct types of relationships: those inherent to the population and those specific to the particular dataset. From a population perspective, relationships observed within a specific dataset may be considered noise or random fluctuations independent of any underlying patterns. NNs can learn both intrinsic and noise-related relationships for a dataset over enough iterations (Zhang et al., 2021). This learning process, while leading to excellent performance on the training dataset, can degrade the model’s ability to predict new data from the same population accurately. This phenomenon, known as overfitting, occurs when an NN learns the noise as if it were a relationship inherent in the population, resulting in misleadingly high performance on the training dataset but poor generalization to the population (Jabbar & Khan, 2015).

To counter overfitting, dataset division is used in NN model training to provide a more accurate estimation of the model’s performance for the population. Typically, a study’s collected dataset is split, with 70%–80% used for training and the remainder for testing (Joseph, 2022). This division is based on the principle that while noises are independent across samples, the inherent population relationship between IVs and DVs remains consistent. Thus, an independent testing dataset serves as a proxy for estimating the NN model’s ability to capture these population-level relationships.

However, this strategy is not without limitations. Given that only about 20%–30% of the dataset is used for testing (Joseph, 2022; Vrigazova, 2021), there is a concern that the small sample size might lead to considerable sampling errors. The limitation in sample size can falsely suggest that IVs can predict DVs in situations where no actual relationship exists (Crockett et al., 2023).

The risk of DE, as previously discussed, becomes more apparent through simulation examples like those provided in the work by Fox and Monette (2024), demonstrating that even when dataset division strategies are employed, methods such as ordinary least squares (OLS) regression can falsely indicate explanatory power in datasets where no actual relationship exists between IVs and DVs. This issue is further compounded in psychological research, which often involves smaller sample sizes. For instance, Zeinalizadeh et al. (2015) highlight a study where only 80 participants were used to estimate an NN model’s performance. Such limited sample sizes significantly increase the risk of sampling errors, which in turn can lead to DEs. In these cases, researchers might incorrectly conclude that IVs can predict DVs based on the misleading performance observed in the testing dataset. This scenario underscores the necessity for careful consideration of sample size and statistical methods to mitigate the risk of drawing inaccurate conclusions from NN analyses.

**Figure 1.**
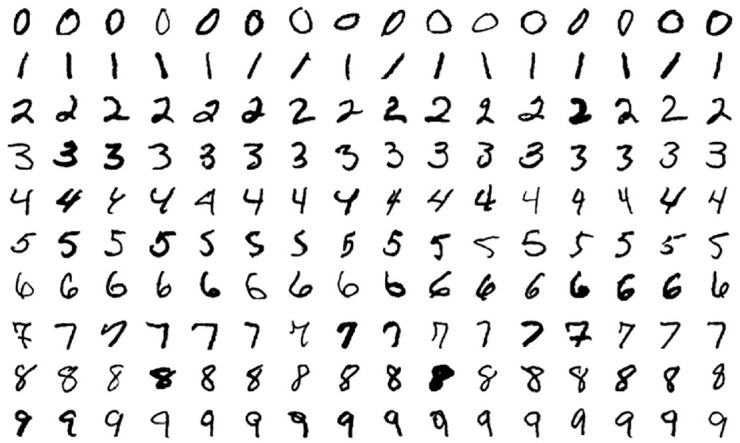
The Modified National Institute of Standards and Technology database (MNIST) Dataset (Deng, 2012) Note: The dataset is the MNIST. Models such as NNs are fitted to recognize handwritten digit images.

Moreover, the DE may often be a blind spot for computer scientists. Computer science researchers mostly deal with datasets where they are sure that there is a true relationship between IVs and DVs, but they are not sure about the forms of relationship between IVs and DVs. For example, there is a true relationship between a figure of a handwritten digit image and the true value of the number in Figure 1. In a scenario like this, all computer scientists need to do is develop a model to recognize the number accurately with the figure of the number. However, psychologists often encounter research questions about continuous or binary DV with a limited sample size (Hullman et al., 2022). In addition, there can be no relationship between IVs and DVs in the population (Wiggins & Christopherson, 2019). For example, whether participants with different personalities have different risks of committing aggressive behavior can be a research question itself (Jiang et al., 2022) before psychologists discuss what kind of accuracy about the risk can be predicted by the personality information of participants.

To our knowledge, there are studies about mislabeled or randomly labeled DV conducted by computer scientists. Although they have reached a consistent conclusion that various kinds of NNs will not provide a DE, their conclusions are based on conditions with multinomial DVs and large sample sizes, and their studies primarily focuses are others, like how these mislabeled multinomial DVs hurt the NN models (Natarajan et al., 2013) or how to use the NN models fitted with random multinomial DVs (Antoniou & Storkey, 2019; Maennel et al., 2020).

To summarize, there exists a theoretical risk that researchers might erroneously conclude a relationship between IVs and DVs based on NN model predictions, falling into DE, especially in instances where no such relationship exists within the population. This concern highlights a significant gap in the current literature, particularly regarding the effectiveness of the training/testing division in mitigating DE risks under limited sample sizes, which will be discussed in the design section. To address this gap and estimate the probability of DE in NN model fitting, our study conducted a Monte Carlo simulation across various sample sizes and examined both binary and continuous DVs. We will apply specific performance criteria that, if met, would lead psychologists to perceive a relationship between IVs and DVs. This approach seeks to provide a more nuanced understanding of DE risks in NN applications, especially in scenarios with limited data.

## Simulation Study

### Design

#### Data Simulation Design

To assess the potential risks of DE in NN, we simulate two types of datasets with several IVs and one DV: (1) Datasets with ordinal IVs and a continuous DV and (2) Datasets with ordinal IVs and a binary DV. As previously mentioned, numerous psychological and educational studies have used the Likert-type scale as an IV for NN model fitting (e.g., Florio et al., 2009; Khan et al., 2019; Marshall & English, 2000; Zeinalizadeh et al., 2015). Therefore, conditions with ordinal IVs are simulated in this study to represent these studies. The entire simulation study is conducted in Python (Pilgrim & Willison, 2009) using the TensorFlow (Abadi et al., 2016) and Keras (Chollet, 2023) packages, with data simulated using NumPy (Harris et al., 2020).

For both types of datasets, IV values are simulated using a discrete uniform distribution with values 1, 2, 3, 4, 5 via the command np.random.choice, with default equal probability choices, i.e., *p* = [.2, .2, .2, .2, .2] for a uniform distribution or *p* = [.05, .1, .2, .3, .35] for a skewed distribution.

Continuous DV values are simulated from a normal distribution *N*(0,1) with the command “np.random.normal.” Binary DV values are simulated from a Bernoulli distribution with *p* = .5 and a Bernoulli distribution with *p* = .1 as the representation for the balanced and unbalanced distribution also with the command “np.random.choice.”

Based on the simulation design, there is theoretically no relationship between the IVs and the DVs in all conditions (Hastie et al., 2009). However, because all algorithms can only provide pseudo-random numbers, it is important for us to ensure IVs simulated in this study cannot be used to predict DV at the population level. To ensure the randomness of this method, we follow the APA simulation study design guidelines (Fan, 2012) by simulating a large dataset to verify the absence of correlations between IVs and DVs.

**Figure 2.**
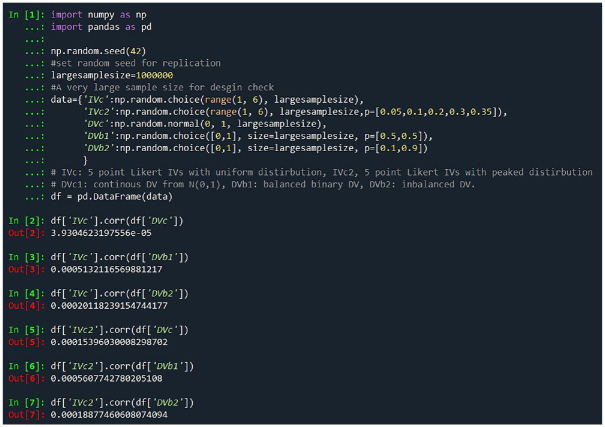
The Check of Random Dataset Simulation With the Large Sample Size Method Suggested by Fan (2012) Note: Checking for the simulated random dataset correlation.

According to the simulation results for a sample size of 1 million, illustrated in Figure 2, there is no linear correlation between any IVs and DVs, confirming the validity of our simulation from a population perspective. We have also done a simulation test, and the code is provided in the supplementary document.

The number of the IV is selected as 3, 5, and 10, which are the conditions we selected from the work by Maxwell (2000). Sample size conditions of 50, 100, 200, and 500 are included in this study. This sample size level is consistent with some psychological studies using NN. For example, Darvishi et al. (2017) have a sample size of 92 + 68 = 160; Allahyari and Roustaei (2022) have a sample size of 380, and Florio et al. (2009) have a sample size of 638. To maximize the proportion of DE, the training/testing dataset division ratios are set as 80:20 in all conditions.

#### NN Design

Psychological studies use various NN designs: Talwar et al. (2022) used one hidden layer with 2 neurons to predict travel intention, Nasser et al. (2019) used one hidden layer with 7 neurons to diagnose Autism Disorder, Zeinalizadeh et al. (2015) used one hidden layer with 30 neurons to predict bank customer satisfaction. Although these NNs are not deep, more studies have not reported the number of hidden layers and neurons they included in their NN model. Compared to the deep models used by computer scientists (Janiesch et al., 2021), the cross-validation procedure on psychometric datasets tends to choose models with fewer neurons, as common relationships between IVs and DV in psychology are usually low-dimensional (Richardson et al., 2017). These three NN design conditions are included in this simulation study. In the meantime, NNs with two hidden layers with 10 neurons in each layer and NNs with two hidden layers with 50 neurons in each layer are included in the study to test if a deeper NN will increase the likelihood of committing a DE. As a result, there are five NN design conditions in total: (2), (7), (30), (10,10), and (50,50). All NNs are forward-propagated, and all layers in NNs are fully connected. (Prechelt, 2012)

To estimate the proportion of DE in NN model fitting, we pretend there is a relationship between IVs and DVs and follow a common NN model-fitting procedure. We use a standard scaler fitted to the training dataset to standardize both the training and testing datasets, enhancing the NN’s performance (Shanker et al., 1996) and ensuring no information from the training dataset is leaked (Rajpurkar et al., 2017).

During model fitting, a portion of the training dataset is randomly selected as the validation dataset. The NN models are then trained on the remaining training data with a maximum of 100 iterations with backpropagation. The performance of the validation dataset is evaluated after each iteration. If there is no improvement over the last 10 iterations, the training stops, and the weights with the best validation performance are used as the final fitted model (i.e., patience is set to 10). The Scaled Exponential Linear Unit (SELU) activation function (Huang et al., 2020) is applied, along with the Adam optimizer (Kingma & Ba, 2014). For a detailed design of the NN model-fitting procedure, please refer to the code in the supplementary document.

#### Criteria

This simulation study requires criteria to establish the minimum predictive performance threshold at which psychologists can confidently conclude a relationship that exists between IVs and DV, thereby committing DE with random datasets. As we have mentioned, the performance of the training dataset is not considered in the performance estimation of the NN model. Besides, many psychological studies using NN have not even reported the model performance on the training dataset. Therefore, all the metrics and criteria are based on the model’s predictive performance on the testing dataset.

Theoretically, this simulation estimates the likelihood that certain patterns between the IVs and the DVs are introduced by sampling error. In addition, similar patterns are present in the validation dataset, leading to the early stopping of the NN training at a reasonable iteration. Furthermore, these patterns also somewhat persist in the testing dataset, providing a certain level of predictive performance.

For the dataset with a continuous DV, the variance that can be explained (i.e., *R*^2^) is commonly used to evaluate and compare the model prediction performance. A variance explained equal to or bigger than 10% was viewed as a minimum acceptable level of prediction performance (Ozili, 2023). Therefore, we establish the following criterion for DE: if an NN model achieves a predictive performance of *R*^2^ ≥ .10, despite no true relationship existing between the variables in the population, we consider this a DE.

In the evaluation of models predicting binary DVs, the area under the curve (AUC) is a commonly employed metric. However, its application is not without challenges, as noted in the summary by Lobo et al. (2008); AUC, which is the area under the curve of receiver operating characteristic (ROC) curve, suffers from several fundamental issues when used as a performance evaluation method on testing datasets. These include its disregard for actual probability values (Ferri et al., 2005), reliance on an aggregate performance measure that may not accurately reflect real-world prediction scenarios (Baker & Pinsky, 2001), and equal weighting of omission and commission errors, despite the varied importance of these errors in different applications (Fielding & Bell, 1997).

Despite these limitations, AUC remains a widely used criterion (Pargent et al., 2023), and as such, we include it as a potential metric for identifying DE in our study. This decision acknowledges the metric’s widespread acceptance, even as we recognize its limitations. Following the discussion on AUC, we will introduce balanced accuracy as an alternative criterion, which will be discussed later. Currently, there is no consensus on the minimum acceptable AUC for psychological studies, highlighting the need for further discussion and potentially establishing more universally applicable criteria.

In evaluating NN model performance, Mandrekar (2010) suggests that an AUC of .7 represents the minimum acceptable level of prediction. However, the psychology field sometimes adopts more lenient criteria, with an AUC of .65 or even .6 deemed acceptable in specific contexts. For example, Epperson and Ralston (2015) consider an AUC of .65 in juvenile sexual recidivism prediction with a significant improvement over chance, equating to roughly a Cohen’s d of .5. Similarly, Doyle et al. (2012) view an AUC of .65 as acceptable for predicting community violence, while Kusuma et al. (2022) accept an AUC range from .6 to .7 for suicidal behavior prediction. In light of these varying standards, our study will evaluate NN models against all three thresholds—.6, .65, and .7—to assess the potential for DE when psychologists interpret these performance levels as indicating meaningful IV–DV relationships. Results for each criterion will be reported separately.

Balanced accuracy offers a different approach to evaluating model performance, especially in contexts where the limitations of AUC significantly affect its utility. Some researchers prefer this metric due to its capacity to provide a less-biased evaluation (Brodersen et al., 2010), especially for binary unbalanced datasets (e.g., Jankowsky et al., 2024; Laufer et al., 2024; Merhbene et al., 2022). Balanced accuracy, the average of sensitivity and specificity, offers an alternative to simple accuracy metrics. Studies such as Belov et al. (2024) and Stamatis et al. (2021), which accept balanced accuracies of .61 and .62, respectively, and Forsell et al. (2020), which proposes a minimum of .65, illustrate the range of acceptable performance levels in the field. Similarly to our approach with AUC, we will apply three balanced accuracy criteria—.6, .65, and .7—to identify DEs, examining the implications of each threshold separately for NN model evaluations in psychological research.

This approach recognizes the variability in performance standards across psychological studies and aims to highlight how these standards may contribute to DEs, thereby enhancing the understanding of model performance evaluation in this field. We discovered that the criteria psychologists use to determine the presence of an effect differ between NN models and traditional NHST. Although NHST makes a binary decision about the null hypothesis, psychologists often require a minimum level of predictive power for an NN model to be considered practically significant. This study takes this difference into account.

With conditions having continuous DV, *R*^2^ ≥ .10 is the only criterion we use. With conditions having binary DV, criteria of *AUC* ≥ .6, *AUC* ≥ .65, *AUC* ≥ .7, *Balancedaccuracy* ≥ .6, *Balancedaccuracy* ≥ .65, and *Balancedaccuracy* ≥ .7 are used.

Based on the design mentioned above, different NN models are fitted with various datasets where there are no relationship between the IVs and DVs. If the model’s performance on the testing data meets or exceeds the criteria, a DE is recorded. However, there are two scenarios in which we assume a researcher would recognize a problem or failure in the model-fitting process and thus would not commit a DE. These scenarios are excluded from the DE estimation. The first exclusion scenario is when an NN model produces identical prediction results for all data points (e.g., all predictions are 0 or 1). In this case, the NN model’s behavior resembles random guessing (Yang et al., 2004). The second exclusion scenario is when all true DV values in the testing dataset are randomly selected to be identical. In this situation, the prediction accuracy can only be estimated for one subgroup, which does not provide meaningful information about the predictive performance of the model.

### Results

#### Results of Conditions With Continuous DVs

Tables 1 and 2 present the results for simulation conditions with continuous DVs. As the results show, the likelihood of committing a DE, where *R*^2^ ≥ .1, is minimal when employing an NN model on a testing dataset with a continuous DV, provided the sample size exceeds 50.

**Table 1.** Result of Continuous DVs on Different Conditions With Uniformly Distributed IVs

Sample size	IV number	NN shape (2)	NN shape (7)	NN shape (30)	NN shape (10,10)	NN shape (50,50)
50	3	0	0	0.001	0.004	0.022
50	5	0.007	0.004	0.01	0.007	0.023
50	10	0.008	0.013	0.016	0.015	0.03
100	3	0	0	0	0	0.004
100	5	0	0	0	0	0.004
100	10	0	0	0	0	0.012
200	3	0	0	0	0	0.001
200	5	0	0	0	0	0
200	10	0	0	0	0	0
500	3	0	0	0	0	0
500	5	0	0	0	0	0
500	10	0	0	0	0	0

Note: The sample size is the overall sample size in the condition, which is the sum of the sample size in the training dataset and testing dataset; IV number is the number of independent variables; under the NN shape (2) is the proportion of result have *R*^2^ ≥ .1 with an NN has 1 hidden layer with 2 neurons; under the NN shape (7) is the proportion of result have *R*^2^ ≥ .1 with an NN has 1 hidden layer with 7 neurons; under the NN shape (30) is the proportion of result have *R*^2^ ≥ .1 with an NN has 30 hidden layers with 2 neurons and under the NN shape (10,10) is the proportion of result have *R*^2^ ≥ .1 with an NN has 2 hidden layers and each have 10 neurons.

**Table 2.** Result of Continuous DVs on Different Conditions With Skewed Distributed IVs.

Sample size	IV number	NN shape (2)	NN shape (7)	NN shape (30)	NN shape (10,10)	NN shape (50,50)
50	3	0.002	0	0	0.001	0.003
50	5	0.005	0.002	0.003	0.009	0.007
50	10	0.013	0.004	0.011	0.015	0.015
100	3	0	0	0	0	0
100	5	0	0	0	0	0
100	10	0	0.001	0.001	0	0.001
200	3	0.002	0	0	0	0
200	5	0	0	0	0	0
200	10	0	0	0	0	0
500	3	0	0	0	0	0
500	5	0	0	0	0	0
500	10	0	0	0	0	0

Note: The sample size is the overall sample size in the condition, which is the sum of the sample size in the training dataset and testing dataset; IV number is the number of IVs; under the NN shape (2) is the proportion of result have *R*^2^ ≥ .1 with an NN has 1 hidden layer with 2 neurons; under the NN shape (7) is the proportion of result have *R*^2^ ≥ .1 with an NN has 1 hidden layer with 7 neurons; under the NN shape (30) is the proportion of result have *R*^2^ ≥ .1 with an NN has 30 hidden layers with 2 neurons and under the NN shape (10,10) is the proportion of result have *R*^2^ ≥ .1 with an NN has 2 hidden layers and each have 10 neurons.

The sample size is the primary factor influencing the proportion of DE. Specifically, a larger sample size correlates with a reduced DE proportion. Other variables, such as NN design and the number of IVs, or whether IV is skewed distributed or not, do not significantly affect DE proportions in the context of continuous DVs.

#### Results of Conditions With Binary DVs

Tables 3 to 6 present findings for balanced DVs with uniformly distributed IVs, imbalanced binary DVs with uniformly distributed IVs, balanced DVs with skewed IVs, and imbalanced binary DVs with skewed IVs, respectively. Despite the differing conditions, the DE outcomes from balanced and imbalanced binary DVs exhibit consistent trends, allowing for a unified discussion except where notable discrepancies arise.

**Table 3.** Result of Balanced Binary DVs on Different Conditions With Uniformly Distributed IVs

Sample size	IV number	NN shape	AUC ≥ .6	AUC ≥ .65	AUC ≥ .7	BA ≥ .6	BA ≥ .65	BA ≥ .7
50	3	(2)	0.284	0.206	0.158	0.319	0.206	0.162
50	5	(2)	0.267	0.178	0.121	0.297	0.178	0.131
50	10	(2)	0.296	0.218	0.153	0.331	0.218	0.162
100	3	(2)	0.191	0.119	0.058	0.216	0.119	0.063
100	5	(2)	0.197	0.111	0.05	0.213	0.113	0.056
100	10	(2)	0.204	0.104	0.048	0.226	0.105	0.053
200	3	(2)	0.12	0.046	0.012	0.125	0.048	0.013
200	5	(2)	0.109	0.04	0.015	0.112	0.041	0.016
200	10	(2)	0.093	0.031	0.005	0.099	0.031	0.007
500	3	(2)	0.036	0.007	0.003	0.037	0.007	0.003
500	5	(2)	0.035	0.006	0.001	0.035	0.006	0.001
500	10	(2)	0.035	0.001	0	0.035	0.001	0
50	3	(7)	0.28	0.217	0.168	0.303	0.217	0.171
50	5	(7)	0.268	0.182	0.131	0.304	0.182	0.137
50	10	(7)	0.269	0.193	0.136	0.3	0.193	0.139
100	3	(7)	0.196	0.108	0.058	0.219	0.11	0.06
100	5	(7)	0.179	0.103	0.038	0.193	0.104	0.041
100	10	(7)	0.194	0.097	0.046	0.207	0.099	0.048
200	3	(7)	0.128	0.048	0.015	0.133	0.048	0.016
200	5	(7)	0.113	0.038	0.015	0.12	0.038	0.015
200	10	(7)	0.109	0.035	0.011	0.114	0.035	0.011
500	3	(7)	0.035	0.008	0.004	0.036	0.008	0.004
500	5	(7)	0.032	0.004	0.001	0.033	0.004	0.001
500	10	(7)	0.021	0.001	0.001	0.021	0.001	0.001
50	3	(30)	0.286	0.212	0.158	0.304	0.212	0.161
50	5	(30)	0.287	0.204	0.158	0.307	0.204	0.161
50	10	(30)	0.261	0.183	0.139	0.291	0.183	0.142
100	3	(30)	0.202	0.125	0.069	0.22	0.126	0.072
100	5	(30)	0.198	0.113	0.056	0.218	0.115	0.058
100	10	(30)	0.193	0.1	0.048	0.214	0.103	0.048
200	3	(30)	0.13	0.049	0.022	0.138	0.049	0.022
200	5	(30)	0.119	0.04	0.009	0.127	0.041	0.009
200	10	(30)	0.138	0.031	0.008	0.142	0.032	0.008
500	3	(30)	0.039	0.008	0.005	0.039	0.008	0.005
500	5	(30)	0.025	0.005	0	0.025	0.005	0
500	10	(30)	0.026	0.005	0	0.026	0.005	0
50	3	(10,10)	0.282	0.21	0.153	0.323	0.217	0.165
50	5	(10,10)	0.285	0.206	0.148	0.317	0.206	0.153
50	10	(10,10)	0.279	0.191	0.136	0.312	0.194	0.142
100	3	(10,10)	0.206	0.129	0.076	0.23	0.135	0.08
100	5	(10,10)	0.208	0.123	0.058	0.224	0.125	0.061
100	10	(10,10)	0.204	0.117	0.051	0.22	0.12	0.052
200	3	(10,10)	0.112	0.04	0.021	0.118	0.041	0.021
200	5	(10,10)	0.106	0.036	0.008	0.115	0.036	0.009
200	10	(10,10)	0.113	0.041	0.008	0.119	0.041	0.009
500	3	(10,10)	0.037	0.006	0.003	0.037	0.006	0.003
500	5	(10,10)	0.023	0.002	0	0.023	0.002	0
500	10	(10,10)	0.024	0.002	0	0.025	0.002	0
50	3	(50,50)	0.261	0.194	0.135	0.301	0.202	0.144
50	5	(50,50)	0.271	0.19	0.151	0.307	0.19	0.156
50	10	(50,50)	0.28	0.19	0.136	0.323	0.192	0.144
100	3	(50,50)	0.2	0.13	0.068	0.223	0.138	0.075
100	5	(50,50)	0.215	0.116	0.052	0.229	0.117	0.059
100	10	(50,50)	0.183	0.097	0.03	0.206	0.1	0.033
200	3	(50,50)	0.128	0.043	0.014	0.133	0.044	0.014
200	5	(50,50)	0.1	0.028	0.009	0.103	0.029	0.009
200	10	(50,50)	0.115	0.034	0.005	0.119	0.034	0.005
500	3	(50,50)	0.044	0.014	0.009	0.049	0.014	0.009
500	5	(50,50)	0.027	0.003	0	0.028	0.003	0
500	10	(50,50)	0.033	0.002	0	0.034	0.002	0

Note: The sample size is the overall sample size in the condition, which is the sum of the sample size in the training dataset and testing dataset; IV number is the number of independent variables; An NN shape of (2) means an NN has 1 hidden layer with 2 neurons; an NN shape of (7) means an NN has 1 hidden layer with 7 neurons; an NN shape of (30) means an NN has 1 hidden layer with 30 neurons; An NN shape of (10,10) means a neural network has two hidden layers and each of them has 10 neurons; an NN shape of (50,50) means an NN has two hidden layers and each of them has 50 neurons. BA means balanced accuracy.

**Table 4.** Result of Imbalanced Binary DVs on Different Conditions With Uniformly Distributed IVs

Sample size	IV numbers	NN shape	AUC ≥ .6	AUC ≥ .65	AUC ≥ .7	BA ≥ .6	BA ≥ .65	BA ≥ .7
50	3	(2)	0.136	0.08	0.04	0.164	0.084	0.048
50	5	(2)	0.126	0.075	0.031	0.173	0.077	0.04
50	10	(2)	0.133	0.085	0.027	0.178	0.086	0.039
100	3	(2)	0.092	0.042	0.02	0.112	0.053	0.032
100	5	(2)	0.107	0.052	0.027	0.12	0.055	0.032
100	10	(2)	0.085	0.037	0.016	0.101	0.039	0.019
200	3	(2)	0.045	0.025	0.019	0.058	0.038	0.032
200	5	(2)	0.076	0.044	0.029	0.079	0.046	0.031
200	10	(2)	0.106	0.062	0.036	0.11	0.066	0.04
500	3	(2)	0	0	0	0	0	0
500	5	(2)	0	0	0	0	0	0
500	10	(2)	0	0	0	0	0	0
50	3	(7)	0.109	0.088	0.049	0.2	0.16	0.124
50	5	(7)	0.135	0.1	0.055	0.174	0.122	0.08
50	10	(7)	0.147	0.105	0.036	0.188	0.117	0.054
100	3	(7)	0.041	0.027	0.017	0.116	0.102	0.092
100	5	(7)	0.077	0.058	0.037	0.135	0.111	0.091
100	10	(7)	0.123	0.08	0.046	0.139	0.089	0.056
200	3	(7)	0	0	0	0.028	0.028	0.028
200	5	(7)	0.004	0.004	0.002	0.016	0.016	0.014
200	10	(7)	0.023	0.02	0.015	0.035	0.032	0.027
500	3	(7)	0	0	0	0	0	0
500	5	(7)	0	0	0	0	0	0
500	10	(7)	0.003	0.003	0.003	0.003	0.003	0.003
50	3	(30)	0.033	0.029	0.018	0.277	0.271	0.26
50	5	(30)	0.066	0.054	0.035	0.279	0.26	0.241
50	10	(30)	0.125	0.101	0.061	0.225	0.185	0.148
100	3	(30)	0.005	0.003	0.002	0.126	0.124	0.123
100	5	(30)	0.01	0.008	0.006	0.107	0.105	0.103
100	10	(30)	0.044	0.031	0.022	0.112	0.097	0.088
200	3	(30)	0	0	0	0.028	0.028	0.028
200	5	(30)	0.003	0.003	0.003	0.015	0.015	0.015
200	10	(30)	0.02	0.02	0.018	0.032	0.032	0.03
500	3	(30)	0	0	0	0	0	0
500	5	(30)	0	0	0	0	0	0
500	10	(30)	0.003	0.003	0.003	0.003	0.003	0.003
50	3	(10,10)	0.039	0.031	0.015	0.759	0.736	0.722
50	5	(10,10)	0.071	0.059	0.035	0.579	0.558	0.535
50	10	(10,10)	0.112	0.089	0.055	0.356	0.314	0.28
100	3	(10,10)	0.006	0.006	0.004	0.953	0.951	0.948
100	5	(10,10)	0.019	0.012	0.011	0.866	0.859	0.857
100	10	(10,10)	0.074	0.063	0.046	0.578	0.565	0.549
200	3	(10,10)	0.001	0.001	0	0.028	0.028	0.027
200	5	(10,10)	0.004	0.004	0.004	0.015	0.015	0.015
200	10	(10,10)	0.028	0.024	0.019	0.039	0.035	0.03
500	3	(10,10)	0	0	0	0	0	0
500	5	(10,10)	0	0	0	0	0	0
500	10	(10,10)	0.009	0.009	0.008	0.009	0.009	0.008
50	3	(50,50)	0.028	0.025	0.015	0.768	0.755	0.746
50	5	(50,50)	0.053	0.04	0.025	0.653	0.629	0.614
50	10	(50,50)	0.096	0.074	0.043	0.405	0.366	0.339
100	3	(50,50)	0.008	0.006	0.004	0.963	0.959	0.956
100	5	(50,50)	0.02	0.017	0.012	0.871	0.867	0.861
100	10	(50,50)	0.06	0.046	0.034	0.566	0.55	0.538
200	3	(50,50)	0	0	0	0.028	0.028	0.028
200	5	(50,50)	0.002	0.002	0.002	0.014	0.014	0.014
200	10	(50,50)	0.03	0.03	0.026	0.043	0.043	0.039
500	3	(50,50)	0	0	0	0	0	0
500	5	(50,50)	0	0	0	0	0	0
500	10	(50,50)	0.003	0.003	0.003	0.003	0.003	0.003

Note: The sample size is the overall sample size in the condition, which is the sum of the sample size in the training dataset and testing dataset; IV number is the number of independent variables, an NN shape of (2) means an NN has 1 hidden layer with 2 neurons; an NN shape of (7) means an NN has 1 hidden layer with 7 neurons; an NN shape of (30) means an NN has 1 hidden layer with 30 neurons; an NN shape of (10,10) means an NN has 2 hidden layers and each of them has 10 neurons; an NN shape of (50,50) means an NN has 2 hidden layers and each of them has 50 neurons. BA means balanced accuracy.

**Table 5.** Result of Balanced Binary DVs on Different Conditions With Skewed Distributed IVs

Sample size	IV number	NN shape	AUC ≥ .6	AUC ≥ .65	AUC ≥ .7	BA ≥ .6	BA ≥ .65	BA ≥ .7
50	3	(2)	0.284	0.215	0.165	0.319	0.215	0.169
50	5	(2)	0.296	0.211	0.158	0.322	0.211	0.162
50	10	(2)	0.276	0.2	0.15	0.306	0.2	0.156
100	3	(2)	0.178	0.095	0.046	0.195	0.096	0.05
100	5	(2)	0.199	0.105	0.055	0.216	0.108	0.059
100	10	(2)	0.177	0.098	0.043	0.198	0.1	0.045
200	3	(2)	0.109	0.034	0.011	0.113	0.034	0.011
200	5	(2)	0.107	0.029	0.01	0.115	0.03	0.01
200	10	(2)	0.115	0.032	0.009	0.12	0.033	0.009
500	3	(2)	0.033	0.006	0.005	0.036	0.006	0.005
500	5	(2)	0.034	0.008	0.003	0.035	0.008	0.003
500	10	(2)	0.027	0.001	0	0.027	0.001	0
50	3	(7)	0.255	0.176	0.128	0.277	0.176	0.13
50	5	(7)	0.274	0.192	0.139	0.3	0.192	0.143
50	10	(7)	0.293	0.216	0.156	0.316	0.216	0.161
100	3	(7)	0.22	0.137	0.07	0.23	0.138	0.072
100	5	(7)	0.192	0.105	0.051	0.211	0.107	0.052
100	10	(7)	0.203	0.115	0.059	0.23	0.118	0.065
200	3	(7)	0.131	0.049	0.017	0.134	0.05	0.017
200	5	(7)	0.111	0.038	0.013	0.114	0.039	0.013
200	10	(7)	0.119	0.037	0.01	0.128	0.04	0.01
500	3	(7)	0.028	0.007	0.003	0.028	0.007	0.003
500	5	(7)	0.029	0.004	0	0.03	0.004	0
500	10	(7)	0.025	0	0	0.025	0	0
50	3	(30)	0.258	0.181	0.135	0.282	0.181	0.139
50	5	(30)	0.293	0.204	0.15	0.315	0.204	0.154
50	10	(30)	0.287	0.195	0.152	0.319	0.195	0.156
100	3	(30)	0.2	0.12	0.063	0.216	0.124	0.066
100	5	(30)	0.212	0.116	0.07	0.226	0.117	0.073
100	10	(30)	0.19	0.099	0.038	0.215	0.101	0.038
200	3	(30)	0.124	0.051	0.027	0.132	0.053	0.027
200	5	(30)	0.107	0.024	0.004	0.109	0.024	0.005
200	10	(30)	0.109	0.035	0.009	0.118	0.035	0.009
500	3	(30)	0.04	0.011	0.005	0.041	0.011	0.005
500	5	(30)	0.04	0.006	0.002	0.041	0.006	0.002
500	10	(30)	0.023	0	0	0.023	0	0
50	3	(10,10)	0.272	0.198	0.142	0.298	0.198	0.147
50	5	(10,10)	0.282	0.202	0.145	0.31	0.202	0.151
50	10	(10,10)	0.274	0.201	0.142	0.297	0.201	0.149
100	3	(10,10)	0.191	0.109	0.042	0.209	0.114	0.045
100	5	(10,10)	0.193	0.126	0.057	0.207	0.128	0.061
100	10	(10,10)	0.18	0.102	0.049	0.205	0.105	0.053
200	3	(10,10)	0.124	0.041	0.017	0.127	0.041	0.017
200	5	(10,10)	0.128	0.042	0.01	0.13	0.042	0.011
200	10	(10,10)	0.12	0.041	0.011	0.129	0.042	0.013
500	3	(10,10)	0.046	0.009	0.003	0.046	0.009	0.003
500	5	(10,10)	0.034	0.003	0.001	0.036	0.003	0.001
500	10	(10,10)	0.025	0.001	0	0.026	0.001	0
50	3	(50,50)	0.277	0.207	0.153	0.306	0.207	0.157
50	5	(50,50)	0.298	0.215	0.153	0.327	0.215	0.156
50	10	(50,50)	0.259	0.178	0.138	0.284	0.178	0.14
100	3	(50,50)	0.199	0.122	0.065	0.215	0.124	0.068
100	5	(50,50)	0.21	0.119	0.054	0.225	0.119	0.054
100	10	(50,50)	0.185	0.096	0.047	0.21	0.097	0.053
200	3	(50,50)	0.114	0.028	0.009	0.12	0.028	0.009
200	5	(50,50)	0.105	0.025	0.008	0.114	0.025	0.008
200	10	(50,50)	0.107	0.042	0.016	0.115	0.043	0.016
500	3	(50,50)	0.039	0.012	0.006	0.041	0.012	0.006
500	5	(50,50)	0.029	0.006	0.001	0.03	0.006	0.001
500	10	(50,50)	0.022	0.001	0	0.023	0.001	0

Note: The sample size is the overall sample size in the condition, which is the sum of the sample size in the training dataset and testing dataset; IV number is the number of independent variables, an NN shape of (2) means an NN has 1 hidden layer with 2 neurons; an NN shape of (7) means an NN has 1 hidden layer with 7 neurons; an NN shape of (30) means an NN has 1 hidden layer with 30 neurons; an NN shape of (10,10) means an NN has 2 hidden layers and each of them has 10 neurons; an NN shape of (50,50) means an NN has two hidden layers and each of them has 50 neurons. BA means balanced accuracy.

**Table 6.** Result of Imbalanced Binary DVs on Different Conditions With Skewed Distributed IVs

Sample size	IV number	NN shape	AUC ≥ .6	AUC ≥ .65	AUC ≥ .7	BA ≥ .6	BA ≥ .65	BA ≥ .7
50	3	(2)	0.147	0.093	0.042	0.205	0.109	0.061
50	5	(2)	0.123	0.08	0.032	0.164	0.085	0.052
50	10	(2)	0.136	0.079	0.024	0.174	0.081	0.032
100	3	(2)	0.099	0.049	0.028	0.124	0.062	0.041
100	5	(2)	0.095	0.048	0.023	0.107	0.05	0.027
100	10	(2)	0.077	0.03	0.015	0.087	0.034	0.018
200	3	(2)	0.065	0.032	0.018	0.074	0.041	0.027
200	5	(2)	0.077	0.054	0.03	0.08	0.056	0.032
200	10	(2)	0.114	0.066	0.031	0.116	0.067	0.032
500	3	(2)	0.001	0.001	0.001	0.001	0.001	0.001
500	5	(2)	0.001	0.001	0.001	0.001	0.001	0.001
500	10	(2)	0	0	0	0	0	0
50	3	(7)	0.112	0.085	0.045	0.199	0.16	0.124
50	5	(7)	0.134	0.092	0.051	0.172	0.116	0.077
50	10	(7)	0.136	0.095	0.04	0.171	0.104	0.052
100	3	(7)	0.041	0.034	0.022	0.113	0.104	0.092
100	5	(7)	0.078	0.054	0.044	0.127	0.102	0.092
100	10	(7)	0.108	0.072	0.036	0.121	0.084	0.05
200	3	(7)	0.003	0.003	0.003	0.021	0.021	0.021
200	5	(7)	0.007	0.006	0.004	0.018	0.017	0.015
200	10	(7)	0.029	0.025	0.021	0.038	0.034	0.03
500	3	(7)	0	0	0	0	0	0
500	5	(7)	0	0	0	0	0	0
500	10	(7)	0.002	0.001	0.001	0.002	0.001	0.001
50	3	(30)	0.028	0.023	0.015	0.283	0.275	0.268
50	5	(30)	0.065	0.052	0.032	0.256	0.241	0.221
50	10	(30)	0.102	0.079	0.046	0.193	0.162	0.133
100	3	(30)	0.003	0.003	0.003	0.104	0.104	0.104
100	5	(30)	0.015	0.014	0.013	0.111	0.109	0.108
100	10	(30)	0.047	0.033	0.025	0.119	0.103	0.095
200	3	(30)	0.002	0.002	0.001	0.02	0.02	0.019
200	5	(30)	0.002	0.002	0.002	0.014	0.014	0.014
200	10	(30)	0.021	0.016	0.014	0.031	0.026	0.024
500	3	(30)	0.001	0.001	0.001	0.001	0.001	0.001
500	5	(30)	0	0	0	0	0	0
500	10	(30)	0	0	0	0	0	0
50	3	(10,10)	0.043	0.039	0.021	0.302	0.295	0.277
50	5	(10,10)	0.067	0.057	0.038	0.237	0.223	0.205
50	10	(10,10)	0.103	0.084	0.04	0.188	0.161	0.119
100	3	(10,10)	0.009	0.008	0.007	0.105	0.103	0.102
100	5	(10,10)	0.017	0.014	0.011	0.103	0.101	0.097
100	10	(10,10)	0.056	0.043	0.031	0.123	0.107	0.095
200	3	(10,10)	0.004	0.004	0.003	0.022	0.022	0.021
200	5	(10,10)	0.003	0.003	0.002	0.014	0.014	0.013
200	10	(10,10)	0.031	0.027	0.024	0.039	0.034	0.031
500	3	(10,10)	0.001	0.001	0.001	0.001	0.001	0.001
500	5	(10,10)	0	0	0	0	0	0
500	10	(10,10)	0.01	0.01	0.009	0.01	0.01	0.009
50	3	(50,50)	0.035	0.024	0.012	0.312	0.299	0.288
50	5	(50,50)	0.05	0.041	0.028	0.257	0.239	0.228
50	10	(50,50)	0.101	0.081	0.05	0.233	0.202	0.173
100	3	(50,50)	0.007	0.005	0.005	0.106	0.104	0.104
100	5	(50,50)	0.025	0.02	0.017	0.12	0.115	0.112
100	10	(50,50)	0.053	0.038	0.028	0.114	0.098	0.088
200	3	(50,50)	0.002	0.002	0.001	0.02	0.02	0.019
200	5	(50,50)	0.005	0.005	0.003	0.016	0.016	0.014
200	10	(50,50)	0.027	0.026	0.023	0.037	0.036	0.033
500	3	(50,50)	0	0	0	0	0	0
500	5	(50,50)	0	0	0	0	0	0
500	10	(50,50)	0.003	0.002	0.002	0.003	0.002	0.002

Note: The sample size is the overall sample size in the condition, which is the sum of the sample size in the training dataset and testing dataset; IV number is the number of independent variables, an NN shape of (2) means an NN has 1 hidden layer with 2 neurons; an NN shape of (7) means an NN has 1 hidden layer with 7 neurons; an NN shape of (30) means an NN has 1 hidden layer with 30 neurons; an NN shape of (10,10) means an NN has 2 hidden layers and each of them has 10 neurons; an NN shape of (50,50) means an NN has 2 hidden layers and each of them has 50 neurons. BA means balanced accuracy.

Simulation results underscore the impact of the AUC and balanced accuracy criteria on DE proportions. Stringent thresholds for these metrics are associated with reduced DE proportions, aligning with our hypotheses and common sense. Moreover, an increase in the number of IVs generally results in lower DE rates. The shape of the NN, whether (2), (7), (30), or (10,10), and the distribution of IVs show negligible differences in DE outcomes. However, an NN configured at (50,50) demonstrates a marginally lower DE rate. This suggests an inverse relationship between the network’s weight count—derived from the number of neurons—and DE proportion.

Notably, AUC-based criteria yield smaller DE proportions than those based on balanced accuracy when compared at equivalent thresholds. Both balanced and imbalanced DV datasets have similar response tendencies to the factors included in the simulation, yet datasets with balanced DVs exhibit higher DE proportions than their imbalanced counterparts. Meanwhile, there is no significant difference in DE proportions between conditions with uniformly distributed IVs and those with skewed IVs included in the simulation studies.

Given these findings, we offer practical guidance for researchers aiming to minimize DE in NN model-fitting endeavors. To achieve a DE rate below .05, a sample size of 500 is advisable when employing balanced accuracy criteria of *≥* .6 or *≥* .65. For a criterion of balanced accuracy ≥ .7, a minimum of 200 samples is necessary, though 500 is preferable for robustness. If a researcher chooses to apply AUC-based criteria, the required sample sizes adjust accordingly: 500 for an AUC of .6, 200 for .65, and more than 100 for .7.

## Discussion, Limitation, and Future Directions

This study estimates the likelihood that researchers mistakenly think that their NN models show a relationship between IVs and a DV when there actually is not one. This study found that for ordinal IVs, a limited sample size with sampling error can create similar patterns that an NN can learn from the training dataset, validate with early stopping, and test on the testing dataset. This occurs even when there is no actual relationship between the IVs and DVs in any of the datasets, and all datasets are independent of each other. Specifically, when the DV is continuous, the chance of committing a DE is pretty low, with sample sizes larger than 50. However, when the DV is binary, psychologists can draw an erroneous conclusion when the sample size is less than 100 or 200 and is subject to different AUC criteria and whether the binary DV is balanced or not.

Based on the Monte Carlo simulation results, this study provides preliminary recommendations for sample size planning when fitting ordinal datasets with NN: a minimum sample size of 500 is necessary to fit an NN model with binary IVs. Unlike previous studies suggesting that the training/testing division can yield highly reproducible results, our study found that outcomes from the training/testing division can still be influenced by sampling error. In addition, the inherently low interpretability of NNs increases the likelihood of researchers committing DEs. If a model’s interpretation is highly inconsistent with established theory, a researcher might suspect the conclusion and replicate the study (Roberts & Pashler, 2000). However, this fail-safe is not applicable to black-box models such as NN (Dayhoff & DeLeo, 2001). From this perspective, this DE estimation simulation study is of unique importance.

This study also highlights the importance of using the right metrics. Metrics such as balanced accuracy rather than AUC should be used in the evaluation of performance when the DV is binary. We have provided some explanations above. Yet, we would also give another explanation based on the training–testing dataset division to prove that the AUC metric should not be used.

The requirement for a specific cut-off point for actionable predictions complicates the use of AUC. This issue is exacerbated when there is no division between training and testing datasets, as is common in many psychological studies (Hullman et al., 2022). AUC metric could be reasonable as this design allows the model to obtain information (e.g., cut-off) from the whole dataset. However, when the training and testing datasets are divided and set as independent from each other in the ML model-fitting procedure, the AUC criterion becomes problematic. In such cases, there would be two AUCs: the AUC provided by the training dataset and the AUC provided by the testing dataset. The former is not reliable due to overfitting, leaving the AUC from the testing dataset as the primary focus. The AUC metric is not a suitable evaluation tool when using a data division strategy where the true labels of the testing set are unavailable. Without knowing the actual values of the DV in the testing dataset, it is impossible to determine the optimal decision threshold for maximizing the model’s accuracy. Furthermore, if we already possessed the DV information for the testing set, there would be no need for prediction in the first place. Therefore, in the context of data splitting, alternative evaluation metrics should be considered instead of the AUC.

Based on the simulation results, we offer practical guidance for researchers aiming to minimize DE in NN model-fitting endeavors. Random data with continuous DV are not very likely to achieve a performance of *R*^2^ ≥ .1 as long as the total sample size is above 50. Yet, to achieve a DE rate below .05 on binary DV, a sample size of 500 is advisable when employing balanced accuracy criteria of *≥* .6 or ≥ .65. For a criterion of balanced accuracy *≥* .7, a minimum of 200 samples is necessary, though 500 is preferable for robustness. Suppose a researcher still wants to apply AUC-based criteria; the required sample sizes adjust accordingly: 500 for an AUC of .6, 200 for .65, and more than 100 for .7. Based on these suggestions, we propose that the studies by Allahyari and Roustaei (2022) and Darvishi et al. (2017) should be replicated, as their limited sample sizes for categorical prediction tasks (i.e., 380 and 92, respectively) put them at risk of DE or inflated predictive performance.

## Limitation and Future Directions

The design suggestion for a reliable qualitative conclusion (i.e., whether there is a relationship between IVs and DVs that can be used for prediction) is just the first step to reaching a good predictive perspective conclusion. This means this study has room for improvement. For example, more non-normal distribution conditions should be included as different non-normal distribution simulation methods can lead to different results (Fairchild et al., 2024). Similarly, continuous IVs should also be included in the simulation. In addition, more NN model-fitting designs should also be included as there are various NN model-fitting designs (e.g., regularization), and the researchers have a high degree of freedom (Donda et al., 2022).

Furthermore, simulation studies should be done on the topic of the NN study design to find a stable quantitative result. Although NN with the predictive conclusion is viewed as a potential solution to the replication crisis in psychology, the result of NN also suffers from replication crisis in the perspective of computer scientists (Bhojanapalli et al., 2021; Laine et al., 2021; Miłkowski et al., 2018). Sometimes, even a difference in random seed choice influences the result (Picard, 2021). Therefore, quantitative psychologists should conduct more simulation studies to provide design suggestions for NN, focusing on the stability of predictive performance. The qualitative empirical evidence found in this study serves as a precaution regarding the instability of quantitative conclusions under these conditions: In scenarios where DE is likely to occur due to sampling error, it is probable that an inflated result can be observed on the testing dataset, even if there is a certain level of relationship between IVs and DVs.

Moreover, our findings on sample size planning for NNs diverge from those in the existing literature. Haykin (2009) suggested a larger sample size was necessary for stable NN model performance, particularly with increased complexity. Conversely, we found that adding layers and neurons to an NN actually increases the risk of DEs. This is likely because simpler models with fewer weights are less prone to fitting noise in the training data. However, we do not see these findings as contradictory. Instead, we emphasize that our study provides empirical evidence to ensure the qualitative results from NNs are reliable, and the required sample size for this may be far less than what is needed for stable quantitative predictions on a testing dataset.

In addition, it should be mentioned that we have provided a contradictory suggestion about sample size planning compared to the literature. Haykin (2009) suggested that a large sample size is required for a stable NN model performance for an NN with more neurons and hidden layers. Yet, we have found that layers and neurons NN increase the probability of committing DE. This is probably because the lower the weight the NN model needs to fit, the easier it can provide the weight that can be used to predict data in testing data by coincidence. Yet, we do not think there is a conflict between these two suggestions. We want to emphasize that we have just provided empirical evidence to ensure that the qualitative result provided by the NN model is reliable, and it is highly likely this sample size is far less than the sample size needed for a stable quantitative suggestion provided by the NN model on the testing dataset.

In light of the high DE proportions found in some conditions of this simulation study, researchers should exercise caution with other performance-based model selection methods. For example, the auto-machine learning approach, which is popular today, involves testing multiple machine learning algorithms on the same dataset and selecting the model with the best performance (Cook, 2016). However, could this design lead to the selection of supervised machine learning models that have a tendency to commit DE? More researches are needed in this direction. Specifically, random datasets should be tested in auto-machine learning methods to gather empirical evidence on model performance under this design.

Regarding performance-based model selection methods, we used hyperparameters (e.g., the number of hidden layers and neurons) as simulation factors. However, these hyperparameters should ideally be determined through cross-validation with grid search (Erdogan Erten et al., 2021). This is a limitation of our study. We did not employ this design for two reasons. First, some psychological studies have also skipped this step and chosen the shape of the NN arbitrarily (e.g., Nasser et al., 2019; Talwar et al., 2022). Second, a Monte Carlo simulation with grid search can be computationally intensive. Although we recommend that future studies use a grid search design, the DE estimation in this study still serves as a valuable reference. This is because performance-based grid search may lead to overoptimization (Gao et al., 2023), which is akin to the DE proposed in this study.

## Supplemental Material

sj-py-1-epm-10.1177_00131644241262964 – Supplemental material for Evaluating The Predictive Reliability of Neural Networks in Psychological Research With Random Datasets Supplemental material, sj-py-1-epm-10.1177_00131644241262964 for Evaluating The Predictive Reliability of Neural Networks in Psychological Research With Random Datasets by Yongtian Cheng and K. V. Petrides in Educational and Psychological Measurement

sj-py-2-epm-10.1177_00131644241262964 – Supplemental material for Evaluating The Predictive Reliability of Neural Networks in Psychological Research With Random Datasets Supplemental material, sj-py-2-epm-10.1177_00131644241262964 for Evaluating The Predictive Reliability of Neural Networks in Psychological Research With Random Datasets by Yongtian Cheng and K. V. Petrides in Educational and Psychological Measurement

sj-py-3-epm-10.1177_00131644241262964 – Supplemental material for Evaluating The Predictive Reliability of Neural Networks in Psychological Research With Random Datasets Supplemental material, sj-py-3-epm-10.1177_00131644241262964 for Evaluating The Predictive Reliability of Neural Networks in Psychological Research With Random Datasets by Yongtian Cheng and K. V. Petrides in Educational and Psychological Measurement

sj-py-4-epm-10.1177_00131644241262964 – Supplemental material for Evaluating The Predictive Reliability of Neural Networks in Psychological Research With Random Datasets Supplemental material, sj-py-4-epm-10.1177_00131644241262964 for Evaluating The Predictive Reliability of Neural Networks in Psychological Research With Random Datasets by Yongtian Cheng and K. V. Petrides in Educational and Psychological Measurement
